# Translating evidence-based guidelines to improve feedback practices: the interACT case study

**DOI:** 10.1186/s12909-016-0562-z

**Published:** 2016-02-09

**Authors:** Karen L. Barton, Susie J. Schofield, Sean McAleer, Rola Ajjawi

**Affiliations:** Centre for Medical Education, Medical Education Institute, University of Dundee, McKenzie Building, Kirsty Semple Way, Dundee, DD2 4BF UK; Division of Food and Drink, School of Science, Engineering & Technology, Abertay University, Kydd Building, Bell Street, Dundee, DD1 1HG UK; Centre for Research in Assessment and Digital Learning, Deakin University, Melbourne City Centre, 550 Bourke Street, Melbourne, 3000 Victoria Australia

**Keywords:** Feedback, Assessment, Self-regulation, Online distance learning, Higher education, Medical education, Dialogue, Postgraduate, Action research

## Abstract

**Background:**

There has been a substantial body of research examining feedback practices, yet the assessment and feedback landscape in higher education is described as ‘stubbornly resistant to change’. The aim of this paper is to present a case study demonstrating how an entire programme’s assessment and feedback practices were re-engineered and evaluated in line with evidence from the literature in the interACT (Interaction and Collaboration via Technology) project.

**Methods:**

Informed by action research the project conducted two cycles of planning, action, evaluation and reflection. Four key pedagogical principles informed the re-design of the assessment and feedback practices. Evaluation activities included document analysis, interviews with staff (*n* = 10) and students (*n* = 7), and student questionnaires (*n* = 54). Descriptive statistics were used to analyse the questionnaire data. Framework thematic analysis was used to develop themes across the interview data.

**Results:**

InterACT was reported by students and staff to promote self-evaluation, engagement with feedback and feedback dialogue. Streamlining the process after the first cycle of action research was crucial for improving engagement of students and staff. The interACT process of promoting self-evaluation, reflection on feedback, feedback dialogue and longitudinal perspectives of feedback has clear benefits and should be transferable to other contexts.

**Conclusions:**

InterACT has involved comprehensive re-engineering of the assessment and feedback processes using educational principles to guide the design taking into account stakeholder perspectives. These principles and the strategies to enact them should be transferable to other contexts.

## Background

Guidelines for improving feedback practices abound. Nicol and Macfarlane-Dick [1] outline seven principles of good feedback including facilitating reflection and self-regulation in learning and encouraging teacher-learner dialogue. Jisc [2] adapted these to six principles to guide assessment for learning design, one being that feedback leads to improvement and stimulates dialogue. Boud and Associates [3] have encouraged curriculum developers and teachers to consider an alternate seven guidelines for improving the educational effect of assessment through engaging the learner, promoting active involvement with feedback, and placing assessment for learning at the centre of subject and program design. In a comprehensive systematic review, Evans [4] distilled twelve educational principles (EPs) of effective feedback and feed forward. However, the difficulty is translating such guidelines into practice.

According to Price and colleagues [5] the current problem with assessment design is a result of oversimplification and poor decision-making. This is despite the availability of guiding principles for assessment and feedback design. For example, a study of teaching staff in a biochemistry course identified that instructors do not change their assessment practices despite more sophisticated design thinking [6]. More worrying is a comprehensive report describing the assessment and feedback landscape in Higher Education in the UK as ‘stubbornly resistant to change’ [7]. Dawson et al [8] argue that to reduce the gap between idealised and real assessment practices, the academic community needs to engage with real, contextualised assessment decision-making. We present the following case study with this in mind.

The original programme, at the Centre for Medical Education, University of Dundee, like many others in Higher Education [9], utilised a monologic information transmission approach to feedback. Providing written feedback on summative assignments at the end of a module meant that as academics we did not know whether our students read, understood and utilised the feedback. Academics spending hours crafting feedback that is not read or used is an inefficient use of time. We needed a better assessment approach that promoted engagement with feedback and encouraged students towards self-regulation [10, 11]. The overall approach we developed was based on students’ self-evaluation of their own work against assessment criteria, written feedback leading to supported reflection on feedback, and student-tutor dialogue [12].

In this paper we showcase knowledge translation in relation to dialogic feedback and explore the challenges and insights gained as a research team which others working with programmatic assessment may find valuable. We present a case study demonstrating how a curriculum development team re-engineered an entire programme’s assessment and feedback practices, in line with good practice recommendations, using action research. A socio-constructivist perspective on feedback is increasingly being encouraged in the literature [11, 13], where learning occurs through student engagement and development of new understandings through dialogue and participation [4]. Stemming from our dissatisfaction with unidirectional written feedback practices, we adopted the perspective that feedback should be a communicative act and a social process [14]. Nicol argues that ‘feedback should be conceptualised as a dialogical and contingent two-way process that involves co-ordinated teacher-student and peer-to-peer interaction as well as active learner engagement’ ([9], pp. 503). The main purpose of feedback we posit is to develop students’ ability to monitor, evaluate and regulate their learning. The interACT (Interaction and Collaboration via Technology) project tackles the problems of monologic feedback transmission and the isolation felt by both students and assessors in an online distance learning programme in medical education [12].

## Methods

The interACT project was implemented within the online Medical Education programme at the University of Dundee’s Centre for Medical Education. The programme enrols between 450 and 500 post-graduate students per year onto its 60 credit Certificate, 120 credit Diploma or 180 credit Masters courses. The courses are made up of 15 credit modules of which the Certificate and the Diploma each have two core modules. The Masters consists of a further 12–15,000 word dissertation. In 2013, the majority of the students on the programme were medics; 54 % were male and 70 % were from the UK and EU.

A case study approach is ideal in this setting where we set out to conduct an in-depth exploration of assessment and feedback practices within a specific context to elucidate the how and why [15]. The methodological design of the interACT project was informed by action research [16], with evaluation being conducted on a continuous basis throughout the duration of the project. Carr and Kemmis define action research as ‘simply a form of self-reflective enquiry undertaken by participants in social situations in order to improve the rationality and justice of their own practices, their understanding of these practices, and the situations in which the practices are carried out’ ([16], pp. 162). There are iterative cycles of planning, action, monitoring and reflection where collaborative wisdom and sharing of information informs the next cycle. At its heart, action research engages in reflexivity, and the research team met regularly during each cycle to reflect on the data collected in relation to the literature and their experiential knowledge. A reference group composed of assessment experts (national and international), an assessment designer in the health professions, higher education and technology experts plus a student representative provided advice and acted as critical friends throughout the lifetime of the project. Two cycles of action research were conducted. A summary of each individual cycle, objectives and research methods used are provided in Table 1. Ethical approval was obtained from the University of Dundee Research Ethics Committee UREC 12024. Written consent was obtained from students and staff who participated in the study.

**Table 1:** Summary of the action research cycles, objectives and methods

Cycle		Timeline	Objective
*Cycle One (01/09/11 to 30/08/12)*			
Phase 1a	Planning problem identification	01/09/11 to 29/04/12	Review of current processes and practice
Phase 1b	Action development of materials and workflow	01/09/11 to 29/04/12	Development and technical testing of interACT
Phase 1c	Monitoring of pilot roll out	30/04/12 to 30/08/12	Piloting of interACT
Phase 1d	Reflection on pilot phase	30/04/12 to 30/08/12	Evaluation and reflexivity
*Cycle Two (01/09/12 to 31/08/2012)*			
Phase 2a	Planning	01/09/12 to 31/12/12	Revision of interACT
Phase 2b	Action	01/09/12 to 31/01/13	Development of modifications to interACT
Phase 2c	Monitoring	01/02/13 to 31/05/13	Implementation of revised interACT
Phase 2d	Reflection	06/06/13 to 31/08/13	Evaluation and reflexivity

### Cycle 1a: Planning problem identification (01/09/11 to 29/04/12)

In the planning phase we conducted a document analysis to better understand the problems associated with assessment and feedback in our programme. A textual analysis was conducted by the authors consisting of: relevant sections from external examiner reports (2006–2011); end of course evaluations (2006–2011); additional evaluation surveys conducted separately in 2010 and 2011 as part of a major curriculum review; and the Postgraduate Taught Student Experience survey. Using a narrative review approach [17] we identified five key problems in relation to assessment and feedback which we sought to address in the interACT project [18]. These were: 1) inconsistency in the quality and quantity of feedback provided; 2) assessment design (e.g., over reliance on essays, limited opportunities for formative assessment); 3) timeliness of the feedback; 4) lack of assessment and feedback dialogue; and 5) isolation of students and tutors.

Having identified these target areas we conducted a literature review focused on feedback (quality, quantity and practices in higher education) and in particular dialogic feedback. Our key aim was to identify research-based evidence and educational theory that would help us address the problems identified through the document analysis. The literature review helped us identify the EPs which we tailored to our context, and five target areas highlighted by the document analysis (in discussion with the reference group). We decided on four EPs that would guide the development of interACT. These were:

1. Feedback should be dialogic in nature
  Feedback is often viewed as something that is ‘given’ to a student to correct their errors, whereas it should be seen as a process of communication which is an on-going evolving dialogue [14, 19]. Simply ‘telling’ a student about their performance does not ensure they have listened (or read the feedback), understood or acted upon it. Feedback should be seen as a social act between individuals, imbued by power, identity and gender, and taking into account respective ideas, feelings and points of view [20]. Feedback dialogues were specifically built into the interACT process.
2. Assessment design should afford opportunities for feedback to be used in future assignments
  Feedback should not be viewed as a single occurrence but as a series of pedagogical opportunities which takes a programmatic approach enabling evidence of learning from feedback to be documented and for feedback to serve to help improve learners’ work in the future [13]. Hattie and Timperley’s [21] model highlights feedforward, related to the question ‘where to next?’, as crucial for learning. Assessment sequencing, formulation of action plans for future work and articulation of how previous feedback informed the current assignment was embedded into the new process.
3. Students should be empowered to seek feedback from different sources
  This principle fits in with capabilities for life-long learning where graduates are required to seek external, credible sources of data to inform their performance and progress [22]. Boud and Soler [23] argue that sustainable assessment (i.e., assessment that promotes lifelong learning) encourages students to make conscious comparisons between their self-evaluations and judgements by teachers, peers and other stakeholders. Research has shown that students make more complex improvements to their work after receiving feedback from multiple sources [24]. Seeking feedback from the tutor on a specific aspect of their work promotes active reflection on the quality of the work, encourages students to define learning goals and prompts the tutor to discuss specific aspects that may not be crucial to the assessment criteria but are important to the student.
4. Feedback should develop evaluative judgements and monitoring of own work
  Learning is enhanced when learners are self-regulating, actively engaged in setting learning goals, selecting strategies for achieving these goals and monitoring their progress toward these goals [1]. Reflecting on feedback and processing it through self-explanation has been shown to improve self-monitoring and evaluation [25]. InterACT prompted students to self-evaluate their assignments before submission against the assessment criteria and in comparison to tutor feedback.

### Cycle 1b: Action development of materials and workflow (01/09/11 to 29/04/12)

With the four EPs and a better idea of the problems faced in our programme we set out designing interACT processes and materials. A full blueprint of the programme assessment was drawn up alongside a description of the standards, criteria for each assessment and the individuals responsible for providing feedback [26]. Assignments were sequenced using the ESCAPE tool [27]. The tool is a simple timeline where formative and summative assessments are represented by different colours to visualise overall sequencing and flow of assignments across an entire programme. Thus the research team were able to analyse the mix of formative and summative assignments and how the assignments were sequenced to enable feedforward (EP 2). The overlap of this project with an ongoing curriculum review enabled the revision of assessment structure to improve sequencing of assignments, to reduce the reliance on essays and to introduce more formative tasks as recommended by several authors in this field [23, 28–30].

**Fig. 1:**
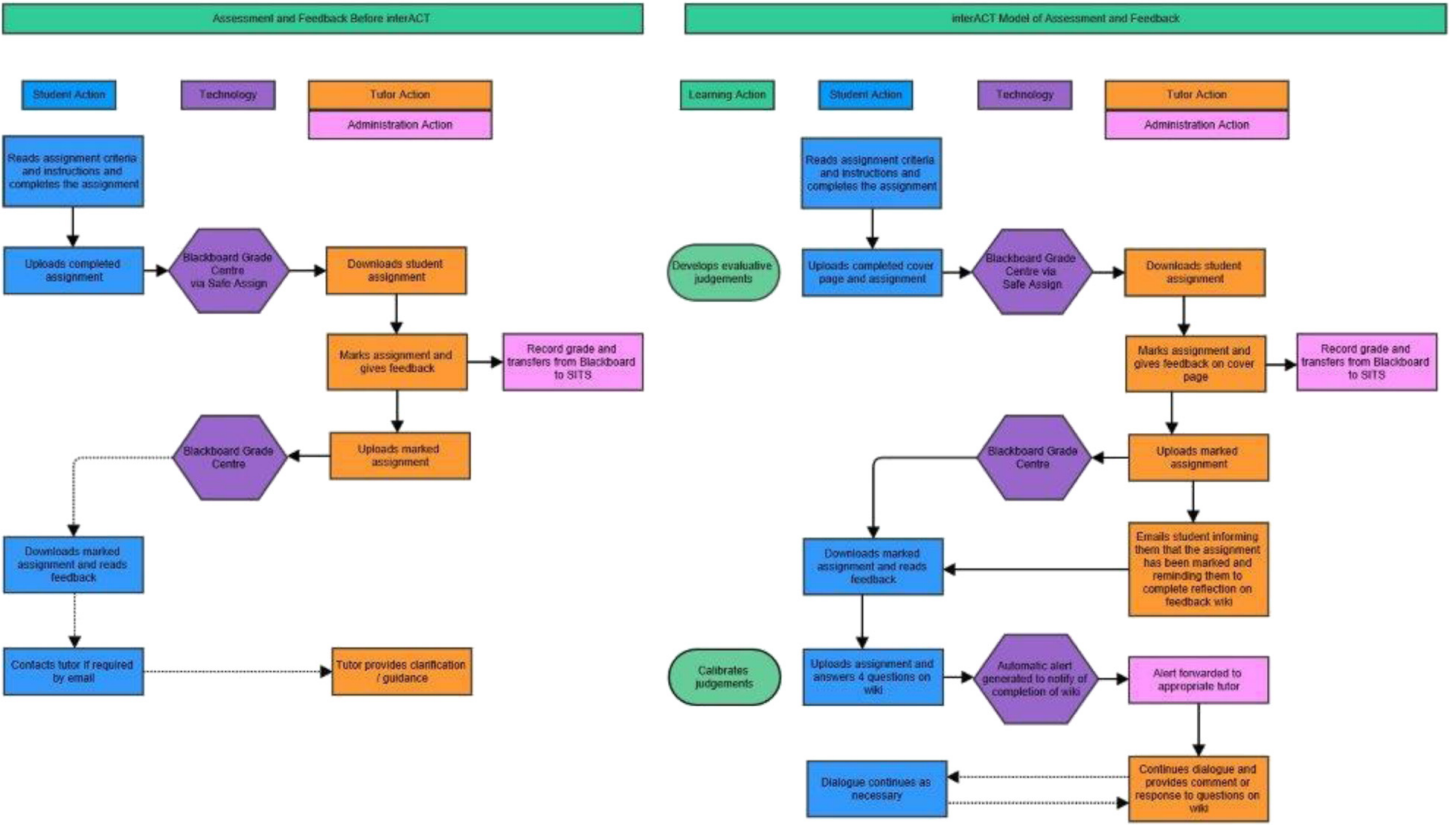
Visual representation of the interACT process for assessment and feedback

The design of the interACT tools and the workflow for the tutor feedback and reflection elements was decided upon through team meetings, focus group with students, communication with the project reference group and discussions among students and staff. The interACT process underwent technical testing (staff and project team members testing the systems with dummy assignments), and adjustments were made where appropriate. The visual representation of the interACT process (Fig. 1) illustrates the assessment and feedback process within the Postgraduate Medical Education Programme before and after the introduction (and revision) of the interACT project.

An individual student feedback journal was created using the Campus Pack™ wiki tool within Blackboard™’s virtual learning environment (VLE). The wiki tool was chosen as it enabled a programmatic repository of assignments (rather than having an individual blog per module). It also allowed an easily navigable portfolio with one page per assignment. The interACT process involved the use of an assignment cover page and self-reflection feedback journal. The cover page is compulsory for all assignments and asks students to review their work qualitatively against the individual assignment’s criteria, request specific feedback if needed, and identify how previous feedback had informed their current work (EP 2, 3 and 4). Tutors provide feedback not only on the assignment but also in response to the students’ self-review, hence commencing feedback dialogue (EP 1). Students then upload their marked assignments into their interACT feedback journal where they answer four questions relating to their interaction with and understanding of the feedback, ensuring students read, critically reflect on and process tutor feedback (EP 4). These were:

1. How well does the tutor feedback match with your self-evaluation?
2. What did you learn from the feedback process?
3. What actions, if any, will you take in response to the feedback process?
4. What if anything is unclear about the tutor feedback?

The tutor is then automatically alerted via email when a student has completed their reflection on feedback journal entry for each assignment. The email contains a direct link to the student’s reflection allowing efficient continuation of the dialogue (EP 1).

### Cycle 1c: Monitoring of pilot roll out (30/04/12 to 30/08/12)

The interACT process was introduced on the 30^th^ April 2012 across the entire programme. Students enrolling on the programme received information about interACT in the induction module. They were educated about feedback and the reflection on feedback process by outlining the research evidence and explaining the alignment between the research principles and the strategies we have used to enact the principles. This process was crucial to establish buy-in from students and help them to get the most out of it. Alongside this we introduced clear instructions and screencasts explaining the process to the students and staff. For 4 months engagement rates with the process were calculated and queries from students were collated to inform the development of an FAQs section to be included with the interACT instructions for students on Blackboard™.

### Cycle 1d: Reflection on pilot phase (30/04/12 to 30/08/12)

After the 4 month introductory phase which comprised the first of the action research cycles, the research team met to reflect on these first 4 months of implementation in terms of workflow and technical difficulties. A number of enhancements were made to the process including: reducing the number of steps required in the process for both students and staff, introducing automatic alerts allowing central management, and informing students by email when their assignments had been marked. The project learning technologist also recorded several screencasts (developed using the Articulate screenr℠) to help direct students in the use of the reflective journal. These were included in the email. Since these changes were made to the process, queries to the administrative team have dramatically reduced, in particular for the most common questions asked relating to using the reflective journal, e.g., how to upload an assignment.

### Cycle 2a: Planning (01/09/12 to 31/12/12)

Figure 1 demonstrates the new model for assessment and feedback, within CME, which is intended to lead to meaningful student-tutor interaction. Re-structuring of the course assessment and feedback process included a reduction in the overall number of assignments, allowing additional time to be spent on feedback dialogue and formative tasks.

### Cycle 2b: Action (01/09/12 to 31/01/13)

The interACT process was streamlined following further development and technical testing of revisions and improvements, and the revised longitudinal feedforward assessment was implemented across the Certificate, Diploma and Masters programmes e.g., an administrator now subscribes to student wikis in order that automatic alerts of any changes to the wiki are generated and these are then forwarded to the appropriate tutor.

### Cycle 2c: Monitoring (01/02/13 to 31/05/13)

During this second cycle attention was focused on evaluating the revised interACT process using a longitudinal transformation mixed methods approach [31] with engagement audit of feedback practices, interviews with students and staff, and a student questionnaire. The evaluation research aims and how the data were collected can be seen in Table 2.

**Table 2:** Evaluation measures

Aim of evaluation	Type of data	Data collection method	Stakeholder
To evaluate engagement with the interACT process: cover page and feedback journal	Quantitative: feedback engagement survey	Feedback engagement audit	Students
To evaluate the impact of interACT on student satisfaction and perceived value to their learning, as well as challenges and enablers to engaging with interACT	Quantitative: impact on workload, satisfaction with interACT Qualitative: Perceptions of improvement in self-review ability, affective feeling of motivation or isolation and recommendations for change	Semi-structured interviews; online survey (via Bristol Online Survey); end of module evaluation report	Students
To evaluate the impact of interACT on staff satisfaction and perceived value to their learning, as well as challenges and enablers to engaging with interACT	Qualitative: experiences with interACT, satisfaction and recommendations for change	Semi-structured interviews; external examiner report	Tutors
To evaluate the impact of interACT on administrative staff satisfaction and workload, as well as challenges and enablers to engaging with interACT	Qualitative: experiences with interACT and nature and number of questions received from students about assessment and feedback	Semi-structured interviews	Administrative staff
To evaluate transferability of the interACT project to the wider HE community	Qualitative: feedback, shared ideas and experiences	Engagement with workshops/ webinar	JISC community

### Engagement audit

Feedback journal entries of all students on the online course were examined and engagement rates with the reflective journal were calculated for three consecutive 4-month periods, one prior to the streamlining changes (4 months into the process) and two following these changes. This was conducted to establish the numbers of students who were participating in the non-compulsory reflection element of the interACT process and to gauge whether rates remained the same as students progressed throughout the programme or decreased due to time pressures, apathy or lack of perceived value.

### Student interviews

Students were invited to participate in the evaluation interviews about the interACT process via email from the project officer. A purposive sample of students at different stages of their studies were selected and interviewed. In-depth semi-structured interviews were conducted by RA and KB with a sample of seven current online distance students to better understand their perceptions of and experience with the interACT process. Questions were asked about: the purpose of feedback; the perceived value of interACT; ease of use; time required; and suggestions to improve design. Interviews were recorded and transcribed. Data analysis was informed by thematic framework analysis [32] starting with reading of the transcripts, negotiation of the thematic coding framework between the two researchers (KB and RA) and coding of the entire data set, followed by developing of themes. The interviews were 25 min on average and ranged from 17 to 37 min in duration.

### Student questionnaire

The themes identified in the interviews were used to inform the development of an online questionnaire, which was sent to all students on the online course who had completed the induction module (*n* = 487). The online questionnaire used Bristol Online Surveys™ and ran from 20th December 2012 to 31st January 2013. The questionnaire included four sections: number of assignments submitted with the interACT process and technical difficulties experienced; reflections on the cover page (positive and challenging aspects); reflections on the journal (positive and challenging aspects); and overall satisfaction with the process. The questionnaire was piloted with postgraduate research students and minor modifications were made to the wording. Students were emailed the link to the survey via their @dundee.ac.uk email address on 20th December with reminders being sent on the 11th and 25th January. Descriptive and thematic framework [32] analyses of the data were undertaken on the quantitative and open-ended questions respectively.

### Staff interviews

In addition 10 staff members (eight tutors and two assessment administrators – all staff involved with the online programme at the time) were interviewed by KB to ascertain their views on the interACT process in terms of their engagement with and whether it encouraged dialogic feedback. Questions were asked on: the purpose of feedback; experiences of interACT; how worthwhile they felt the process was; and suggestions on how to improve engagement with students. Data analysis was informed by framework analysis [32] starting with reading of the transcripts, negotiation of the thematic coding framework and coding of the entire data set, followed by developing of themes by KB and RA . The interviews were 35 min on average and ranged from 22 to 43 min in duration.

### Cycle 2d: Reflection (01/06/13 to 31/08/13)

Data from Cycle 2 of the project were compared with data from Cycle 1 to determine the impact of the revisions to our assessment and feedback process. Expert advice was sought from members of the reference group regarding the findings of the project and future sustainability. The reflection process highlighted the need for an educational package to be developed for students and staff to improve assessment literacy (discussed below).

## Results

### Engagement surveys

Since the implementation of the interACT process, following a 4 month introductory period, 100 % student completion rates have been achieved with the compulsory addition of the cover page to each assignment. The reflective journal component, although not compulsory, has been strongly encouraged and its use has been remarkably high. Engagement with the reflective journal increased as a result of streamlining the process (as described above) and queries to the administrator about the process reduced to a couple per month. Table 3 shows the improvement in engagement after enhancing the process in the second period of measurement and stabilisation in the third period for the certificate core modules (31–88 % before, 52–87 % second and 58–77 % for the third period). This demonstrates a key learning point that the ease of use of any assessment and feedback processes is crucial for getting students and staff to engage with the process.

**Table 3:** Students Engaging with the Reflective Journal Feedback Process

Module	Assignment No	30th April to 31st Aug 2012			1st Sept to 31st Dec 2012			1st Jan to 30th April 2013		
		Submitted	Reflective journal	%	Submitted	Reflective journal	%	Submitted	Reflective journal	%
Induction		163	112(9)	68.7	155	100(2)	64.5	185	108(6)	58.4
Teaching and Learning	1	34	30	88.2	63	48	76.2	62	37(1)	59.6
	2	27	20(1)	74.1	39	30	76.9	64	44	68.8
	3	30	22	73.3	34	29	85.3	52	33	63.5
	4	25	16(1)	64.0	37	32	86.5	48	33	68.8
Principles of Assessment	1	23	14	60.9	31	21(2)	67.7	43	31	72.1
	2	16	5	31.3	29	21	72.4	39	30	76.9
	3	16	6	37.5	29	24(1)	82.8	37	25	67.6
	4	9	3	33.3	29	15(1)	51.7	40	25	62.5

Numbers in brackets in the Reflective Journal columns are those who have uploaded their assignment to the feedback journal but not engaged in dialogue and are not part of the total count

### Student interviews

Responses from students were mostly positive with students commenting on the value of the interACT process to their learning. The students reported valuing the structure provided by the cover page and the opportunity to reflect on what they had done relative to the assessment criteria. They also valued the opportunity for dialogue with staff about their work brought about by the reflective journal.

#### Purpose of feedback

Students were aware that feedback should be more than just about the process of correction and that it should provide direction both for the assignment and for future study. This reconceptualisation of feedback to promote self-regulation was fundamental to the InterACT approach:

*The purpose of feedback is to help regulate my own learning decisions, as I understand it, and to give me some idea of where I am going in relation to where I am supposed to be going and help me adjust my decisions and my learning efforts to meet those goals.* Interview Student 4

#### Structure of feedback process

Overall they appreciated how the cover page and the reflective journal provided a structure to the feedback process:

*It gives a structure to the feedback doesn’t it, otherwise you just ask ‘what do you think about the assignment?’ you’re just going to get ‘it was Ok’ from most people.* Interview Student 7

#### Reflection and learning

The cover page prompted a change in students’ approaches to their assignments through prompting (further) evaluation of their work, providing the opportunity to reflect on and review their work before submitting:

[interACT] *did force my reflective process in the end to have one last look without changing anything on my self-evaluation, it is not something that I think I would have naturally done.* Interview Student 3

Students also commented on the value of the prompted questions in the cover page and reflective journal encouraging learning from the feedback. The first quote below highlights how the process has prompted explicit thought on feedforward, while the second quote refers to a sense of empowerment with being able to ask for specific feedback that meets individualised learning goals:

[With interACT] *you are then a bit more mindful of what your feedback was from the first one [assignment] to any changes that you might make in the subsequent assessments. I suppose in a way before you would just think about your answer a bit and the feedback that you have got but not writing it and writing it down makes a difference.* Interview Student 6

*What I really like about it, is that it gives me a chance to request, to maybe direct a little bit the specificity of what kind of information I am going to get … I got quite a bit of specifics [feedback] about that which allowed me to change what I did. I think it gives me the chance to work on what I define.* Interview Student 3

#### Feedback dialogue

The majority of students agreed that the interACT process promoted feedback dialogue, something particularly important in the sometimes isolating context of online distance learning, which they saw as a positive thing. Feedback dialogue was also seen to strengthen relationships between tutors and students and ‘personality exchange’:

*I have been able to engage in more of a dialogue with whoever has graded it, which has allowed a little more personality exchange and a little more support, when you feel that there is someone at the other end actually looking at what you are working so hard at and treating you as a person, because the challenge for both of us I think is that we don’t get to have face-to-face so there is no laughing over a cup of coffee.* Interview Student 4

#### Recommendations

Despite the cover page being tailored to each assignment, the students were critical of the repetitive nature of the cover page and reflective journal and felt that they needed to be more specific to each assignment:

*For things like style, format and language, I find that I am putting the same thing for all of them because I don’t think that there has been a big change in my style, format and language across the different essays.* Interview Student 1

### Student questionnaires

Fifty-four students (11 %) completed the questionnaire. Of these, 46 (85 %) thought that the instructions provided about the assignment submission process (cover page, uploading marked assignment and reflecting on the journal) were clear. Respondents found that the process of self-review and reflecting on feedback was valuable for their learning (52 %); promotes assessment and feedback dialogue (48 %); and promotes self-evaluation (63 %). Students were evenly split on whether they agreed or disagreed that the process was time-consuming.

The qualitative comments re-iterated the interviews with students reporting the value of being able to ask for specific feedback, providing an opportunity for final review before submission, a chance to express their views and a structure for submission and feedback expectations. Three challenges were reported with the process (one was related to the size and formatting, another to repetitive nature of the reflection and third related to lack of staff engagement with the students’ comments).

### Staff interviews

Overall tutors rated the interACT process positively. Initial concern about the additional time involved did not prevent tutors from engaging as the value and satisfaction from engaging with learners and seeing their feedback make a difference to student learning negated the extra 5–10 min per assignment reported to be needed.

*Initially, I probably thought, thought this is going to be a lot of work, and it is a lot of work but I think it’s beneficial to the students so it is money well spent in that sense and I think the student does get a lot out of it.* Interview Tutor 3

*I have had some students comment this back to me, that it is really good for them to feel that they have a dialogue with the tutor.* Interview Tutor 2

#### Purpose of feedback

There was evidence of repeated cycles of dialogue on the cover page and reflective journal (within and across assignments) that contributed to learning. All of the tutors conceptualised feedback as being more than just about correction, highlighting buy-in with the EPs underpinning interACT:

*Feedback has to go beyond just correcting students’ mistakes. I think it really does have to develop their evaluative judgements capacities. So their ability to evaluate their own work, to seek feedback about it, to monitor and judge and then to develop action plans to address any identified learning needs. So it has to be about helping them to self-regulate their learning.* Interview Tutor 1

*Feedback should be all about helping the student to identify what they’re doing well, to identify what they need to further develop and to support them in that development.* Interview Tutor 2

Tutors’ views and experiences of interACT were positive in terms of what the project was trying to achieve in relation to the interACT EPs.

*I think it’s overall positive. What I think is good about them, I think it’s good that it does prompt self-evaluation. I think it’s good that it gets students to think about what sort of feedback they want because that’s what we are training them to do, that’s what they should be doing after they graduate is seeking feedback. That’s what we do to calibrate our performance.* Interview Tutor 1

#### Structure of feedback process

Tutors found that the structured way that feedback is now given was positive. The cover page led to changes in how some of the tutors approached feedback in terms of structure and the amount of feedback that they are giving.

*It encourages you to provide lengthy feedback I think and certainly the way in which, because when I in a previous job we had cover sheets but they were tick boxes then we had to make three points for improvement and it was very narrow whereas this gives you much more options to be more personalised with the feedback.* Interview Tutor 7

#### Reflection and learning

An unexpected benefit was that some staff members reported an improvement in their own feedback processes as the categories prompt them on the assessment criteria and what they should be providing feedback on.

*It’s certainly improved my feedback in terms of definitely quantity and hopefully quality as well so I’m giving more information now because I’m being constantly prompted.* Interview Tutor 3

Tutors reflected that the feedback they were giving had improved and valued the feedback they themselves received from learners for their own development. They also commented that the reflective journal has been a simple way of closing the feedback loop because they receive feedback on the assignment and if anything is unclear (either regarding the assessment instructions or the feedback information) they can address this quickly. They appreciated the ability to quickly respond to a student highlighting a problem with the module:

*I also use it for closing the feedback loop so if they tell me something’s wrong or they don’t understand it then I will also use that to say ‘thank you for highlighting that, I have now changed it’.* Interview Tutor 2

#### Feedback dialogue

The question on the cover page prompting students to highlight specific areas for feedback was viewed as the most important by some of the tutors as it enables greater personalisation of feedback and meeting of student needs. This along with the reflective journal created dedicated spaces for students to come forward and ask questions about the feedback that they had received and led to increased dialogue with the distance learning students which was overwhelmingly viewed as positive:

*Yeah definitely I think that* [it’s encouraging dialogue]*, I just even think that the fact that you’re able to go ‘yeah I agree with you’ or ‘well no actually this is better than you think it is’ is a dialogue that you would never have had, it’s all one direction feedback in the past.* Interview Tutor 7

#### Recommendations

Academic staff recommended improvements to the process - most viewed the cover page process more favourably than the reflective journal, which was thought to be “a bit clunky”. Further streamlining with alerts and automatic upload of assignments into the reflective journal were recommended. Several tutors discussed how the cover page could be better tailored to the assessment to avoid any repetition and suggested improvements to the reflective journal. In addition, there was scope for student and faculty development in critical reflection and evaluation of work:

*We need to include more guidance to the students on how to engage with the cover page … it would help them if they had some examples of good engagement and good self-review because one of the reasons we’re putting this in is to develop their self-review skills,* [also] *we need faculty training on that about how do we develop their self-review skill.* Interview Tutor 2

### Administrative staff

Administrative staff found that they were receiving less queries about assessment and feedback from students, which they attributed to the feedback being more thorough and structured:

*I would say* [I get] *less* [questions]*, I think just because the feedback is more thorough.* Interview Administrator 2

They commented that the introduction of the cover page has reduced their workload when administering a re-submitted assignment as all the feedback was now in one document:

*It’s a lot easier for those ones that I have to do the resubmissions on because the feedback is already incorporated in the attached area. I’m not having to do any copy/pasting from the original.* Interview Administrator 1

They also felt that the number of technical queries had sharply declined following the development of the screencasts, streamlining of the process and the introduction of the email alerting students to their assignment being marked.

## Discussion

Several key findings have emanated from this project. First it is possible to translate educational guidelines and principles to inform practice specifically for feedback in an online distance learning programme, although this is not without its challenges. Changes in the assessment and feedback processes and specifically the tools (e.g., the cover page questions) resulted in positive changes in the way students and staff approached feedback. The cover page prompted an added level of evaluation against the assessment criteria and learning objectives and also encouraged students to seek specific feedback. The questions in the reflective journal prompted reflection on feedback, explication of actions for future assignments and encouragement of student-tutor dialogue. The cover page prompted staff to structure their feedback practices in accordance with the assessment rubric and to tailor feedback to students’ specific requirements. The reflective journal provided instant feedback on what students had understood from the feedback and what remained unclear, hence closing the evaluation loop. Despite the additional time requirement with interact staff were keen to see the process embedded into the programme, overall efficiency was managed by reducing the total number of summative assessments (shifting efforts from assessment *of* learning to assessment *for* learning).

Bloxham and Campbell [33] used interactive cover pages to promote feedback dialogue between students and tutors. They found that their students were not experienced enough to know what was expected and to initiate meaningful dialogue with their tutors. In our study, some students utilised the opportunity to seek feedback on specific aspects of their work very well, while others either ignored the question or sought feedback on ‘all of the assignment’. This is an area where students’ assessment literacy may be improved. As with Crimmins et al [34] who developed a written, reflective feedback process for first year undergraduate student, we found the dialogic process supportive of relationship building. Our students talked about ‘personality exchange’ and opportunity to ‘interact freely’ with the staff. This was important for us as students and staff had reported feeling isolated. Furthermore, the influence of the educational alliance on feedback receptivity is a concept starting to be explored in the literature [35] and based on our findings is clearly something valued by students and staff. Engagement with the feedback in the cover page and reflective journal was a particular strength of the design.

Streamlining the process was key to improving student and staff engagement, highlighting the importance of getting buy-in and evaluation from all stakeholder groups. Despite the increased time it takes for staff to engage with the feedback dialogue, all staff wanted to continue with it. This is due to perceived improvement in their own feedback practices, closing the evaluation loop, perceived value for student learning and increased tutor satisfaction in engaging with learners and seeing their feedback used. The time required by the tutor to engage with interACT has been offset by reducing the overall number of assignments that students complete on the programme. Based on the findings from the staff interviews we would advocate for a further EP to underpin interACT, that the feedback process should be used to improve teaching [1, 2].

Although engagement has been improved quantitatively, the project team would like to further improve the quality of students’ assessment literacy, evaluative judgment and their feedback-seeking ability. It is planned to tackle this through educational activities in the first core module of the programme and through feedback on the learners’ self-evaluation. Engaging in the process of providing peer review is also beneficial for developing evaluative judgement [36] and ways of embedding peer feedback into a non-cohort online distance learning programme are being investigated. However, the affordance of such a programme where there are no arbitrary assessment deadlines, is that students can take the time and effort needed to reflect on and address their formulated action points before submitting the next assignment, hence strengthening the feed forward element [10]. To make the most of the process it is planned to introduce a patchwork assessment [37] in which students identify how they have developed across the programme to meet exit outcomes of the Certificate and Diploma programmes using evidence from their reflective journal. In this way it is believed that students will gain from further reflection on their feedback dialogues as well as it being an efficient way to assess the reflective journal.

## Strengths, limitations and future research

The strong theoretical underpinning, multiple methods adopted and repeated cycle of action research are strengths. Participation in the student questionnaire was low but perhaps not surprising given that our students are busy health professionals studying at a distance. Although enrolled on the course, some of the students may not have yet submitted any assignments when the questionnaire was released and so the reported response rate is potentially lower than the actual one. Changes in evaluative judgements were not measured as students progressed through the course; this would be an interesting area to research. Further testing of the interACT process within other programmes would be important. The project team are currently conducting an interactional analysis of the feedback dialogue excerpts to shed light on how these are constructed and negotiated and how learning is mediated through feedback dialogue in an online context [38].

## Conclusions

InterACT has involved comprehensive re-development of the assessment and feedback processes using EPs translated from the literature to guide the design keeping in mind the project stakeholders. These principles and the strategies to enact them should be transferable to other contexts. The action research cycles have enabled further refinement to the interACT process, to develop FAQ sections to assist students, and to provide examples of areas students ask for specific feedback on. This could be used to provide group feedback to future students, thus increasing tutor efficiency. Evaluation findings have been used to inform faculty development and will continue to be used to make improvements to the interACT process and for student and faculty development.
